# Chitin-enriched insect frass fertilizer as a biorational alternative for root-knot nematode (*Meloidogyne incognita*) management

**DOI:** 10.3389/fpls.2024.1361739

**Published:** 2024-03-05

**Authors:** James Kisaakye, Dennis Beesigamukama, Solveig Haukeland, Sevgan Subramanian, Paul K. Thiongo, Segenet Kelemu, Chrysantus M. Tanga

**Affiliations:** ^1^ International Centre of Insect Physiology and Ecology (icipe), Nairobi, Kenya; ^2^ Division of Biotechnology and Plant Health, Norwegian Institute for Bioeconomy Research (NIBIO), Ås, Norway

**Keywords:** insect exuviae, insect frass, organic chitin, nature-based solution, bionematicide

## Abstract

Root-knot nematodes (*Meloidogyne* spp.) are serious pests of most food crops, causing up to 100% yield loss. Nevertheless, commercial nematicides are costly and harmful to the environment. While the nematicidal potential of crustacean and synthetic chitin has been demonstrated globally, research on the potential of insect-derived chitin for nematode control has received limited attention. Here, seven chitin-fortified black soldier fly frass fertilizer extracts (chFE) were assessed for their suppressiveness of *Meloidogyne incognita* and impacts on spinach growth in comparison with a commercial nematicide using *in vitro* and *in vivo* bioassays. The performance of chFE and control treatments was assessed by determining their effects on nematode egg hatchability; infective juvenile (J2) mortality and paralysis; number of galls, egg masses, and J2s per plant; and spinach root and shoot biomass. *In vitro* results showed that chFE and commercial nematicide suppressed nematode egg hatchability by 42% and 52%, respectively, relative to the control (sterile distilled water). Up to 100% paralysis was achieved when *M. incognita* J2s were exposed to either chFE or commercial nematicide. Further, the J2 mortality achieved using chFE (95%) was comparable to the value achieved using commercial nematicide (96%); in all treatments, mortality increased with exposure time. Similarly, up to 85% suppression of gall development was achieved when spinach plants were grown in soil drenched with chFE; up to 79% reduction in egg mass formation and 68% suppression of J2 development in the root system were achieved using chFE. Also, chFE application significantly increased spinach root and shoot biomass by 54%–74% and 39%–58%, respectively, compared to commercial nematicide. Our findings demonstrate the nematicidal potential of chFE and its benefits on crop production. Thus, chFE could be considered as a promising multipurpose, regenerative, and cost-effective input for sustainable management of plant-parasitic nematodes and enhancement of crop yield.

## 1 Introduction

Root-knot nematodes (RKNs; *Meloidogyne* spp.) are a polyphagous group of plant-parasitic nematodes that parasitize most, if not all, cultivated crops. While RKNs have global economic importance, their impact is greater in the tropics (Maurice et al., 2010; Sikora et al., 2018). The four most important species are the tropical *M. incognita*, *M. arenaria* and *M. javanica*, and the temperate *M. hapla* (Maurice et al., 2010; Onkendia et al., 2014). *Meloidogyne incognita*, *M. javanica* and *M. hapla* possess the highest rate of global spread among other crop pests and pathogens (Bebber et al., 2014), with *M. incognita* reported to be the most damaging within the genera (Maurice et al., 2010; Ralmi et al., 2016). The second stage juvenile (J2) is the infective stage, which penetrates the root, usually behind the tip, and moves through the root to establish and develop a lifelong feeding site. The feeding site contains a few metabolically active multi-nucleate giant cells, located close to the vascular bundles, and provides constant nourishment to the developing female (Abad et al., 2010; Jones and Goto, 2011). Consequently, water and nutrient uptake by the host plant is substantially reduced, leading to compromised plant growth and a significant reduction in yield (Wesemael et al., 2011; Coyne et al., 2018a). Up to 100% yield loss has been attributed to RKN infection, especially in high-value crops, which manifests as a decline in the quality and/or quantity of the produce, and increased production costs (Trudgill et al., 2000; Wesemael et al., 2011; Onkendia et al., 2014). Disease complexes with other pathogens such as *Ralstonia solanacearum*, *Rhizoctonia solani*, *Fusarium oxysporum* species complex, and *Thielaviopsis basicola* have also been reported (Manzanilla-López and Starr, 2010), an indication of the continued need to develop more effective and sustainable management strategies against this pest.

Several methods have been recommended for the management and control of RKN infection; these include the use of botanicals and chemical nematicides, crop rotation, resistant varieties and biological control (Perry et al., 2010; Onkendia et al., 2014; Ralmi et al., 2016). Traditionally, farmers have mostly relied on the use of chemical nematicides like the soil fumigant methyl bromide, while a majority of farmers, especially in the tropics, practice farming without implementing any RKN management strategies. Unfortunately, the use of most chemical nematicides has been discouraged or banned due to growing concerns about human, animal and environmental safety (WHO, 2020), requiring alternative user- and eco-friendly management strategies.

Chitin is a natural polysaccharide that occurs as a structural component in most insect pupal exuviae and crustacean shells. However, chitosan is a deacetylated derivative of chitin. Deacetylation of chitin to chitosan can be achieved through chemical or biological means (Schmitz et al., 2019; Santos et al., 2020). Both chitin and chitosan have gained attention for use in different sectors including agriculture (Sharp, 2013; Grégorio and Lichtfouse, 2019; Shamshina et al., 2020; Kemboi et al., 2022). This is attributed to their exceptional properties, which include biodegradability, biocompatibility, non-toxicity and antimicrobial activity (El Knidri et al., 2018; Lagat et al., 2021).

The black soldier fly (BSF; *Hermetia illucens*) is a two-winged wasp-like fly belonging to the family Stratiomyidae. It is native to the tropics and has spread to the subtropical and temperate regions of the world (Diclaro and Kaufman, 2012). The fly undergoes complete metamorphosis, with the entire lifecycle lasting between 40 and 43 days (Tomberlin et al., 2002). The BSF larvae are efficient bio-waster recyclers, capable of consuming all types of organic waste. As they feed, the BSF larvae leave behind a mass of excreta and unconsumed organic matter, collectively referred to as frass (BSFF), which contains a substantial amount of nutrients (Lalander et al., 2015). After approximately 14 days of feeding, the larvae pupate and turn into dark-colored non-feeding pupa covered in an exoskeleton, from which the adult fly emerges, leaving behind the chitin-rich dark-colored pupal shell, also called BSF exuviae (BSFE).

The chemical extraction of chitin from BSF exuviae requires the use of bases and acids such as sodium hydroxide and hydrochloric acid for the deproteinization and demineralization processes, respectively (Radwan et al., 2012; Abidin et al., 2020). However, the acids and bases used for chemical extraction are costly, hazardous to the environment, non-biodegradable and can affect the physicochemical properties of the final product (Tan et al., 2020). Conversely, microbial extraction of chitin from BSF exuviae has been demonstrated, and the concentrations have been determined. Moreover, in addition to utilizing eco-friendly microbes, the method compromises neither the quality nor quantity of the extracted chitin (Lagat et al., 2021).

While the nematicidal potential of crustacean and synthetic chitin has been studied (Radwan et al., 2012; Mota and dos Santos, 2016; Zhan et al., 2021), information regarding the potential of using insect-derived chitin for nematode control is very limited. Thus, this study was conducted to assess the potential of microbially extracted chitin from BSF (*H. illucens*) frass and pupal exuviae against the root-knot nematode *M. incognita* under *in vitro* and greenhouse conditions.

## 2 Materials and methods

### 2.1 Chitin-fortified frass fertilizer extracts

Black solder fly frass and BSFE, generated from BSF, effective microorganisms (EMs), biochar and molasses, were used in the fermentation process to generate chitin-fortified frass fertilizer extracts (chFE). Black soldier fly frass and BSFE were obtained from a BSF colony maintained at the Animal Rearing and Containment Unit (ARCU) of the International Centre of Insect Physiology and Ecology (*icipe*), Nairobi, Kenya. The BSFs were reared on a mixture of potato peelings, fruits and vegetable waste, following a standard rearing procedure (Shumo et al., 2019; Beesigamukama et al., 2021). The BSFF obtained was milled into a fine powder (≤0.5 mm) using a hammer mill. The BSFE were washed in tap water, sun-dried to achieve a moisture content of ≤10%, and milled into a fine powder (≤0.5 mm). Biochar was obtained from Safi Organics Ltd., Mwea, Kenya, while molasses was sourced from an agrovet shop in Thika, Kenya. The EM culture was sourced from EM Technologies Ltd., Embu, Kenya, and contained the following active microorganisms: lactic acid-producing bacteria; *Lactobacillus plantarum* and *Lactobacillus casei*, photosynthetic bacteria; *Rhodopseudomonas palustris*, protease-producing bacteria; *Pseudomonas aeruginosa* and *Bacillus subtilis*, yeast; and *Saccharomyces cerevisiae*.

**Figure 1:**
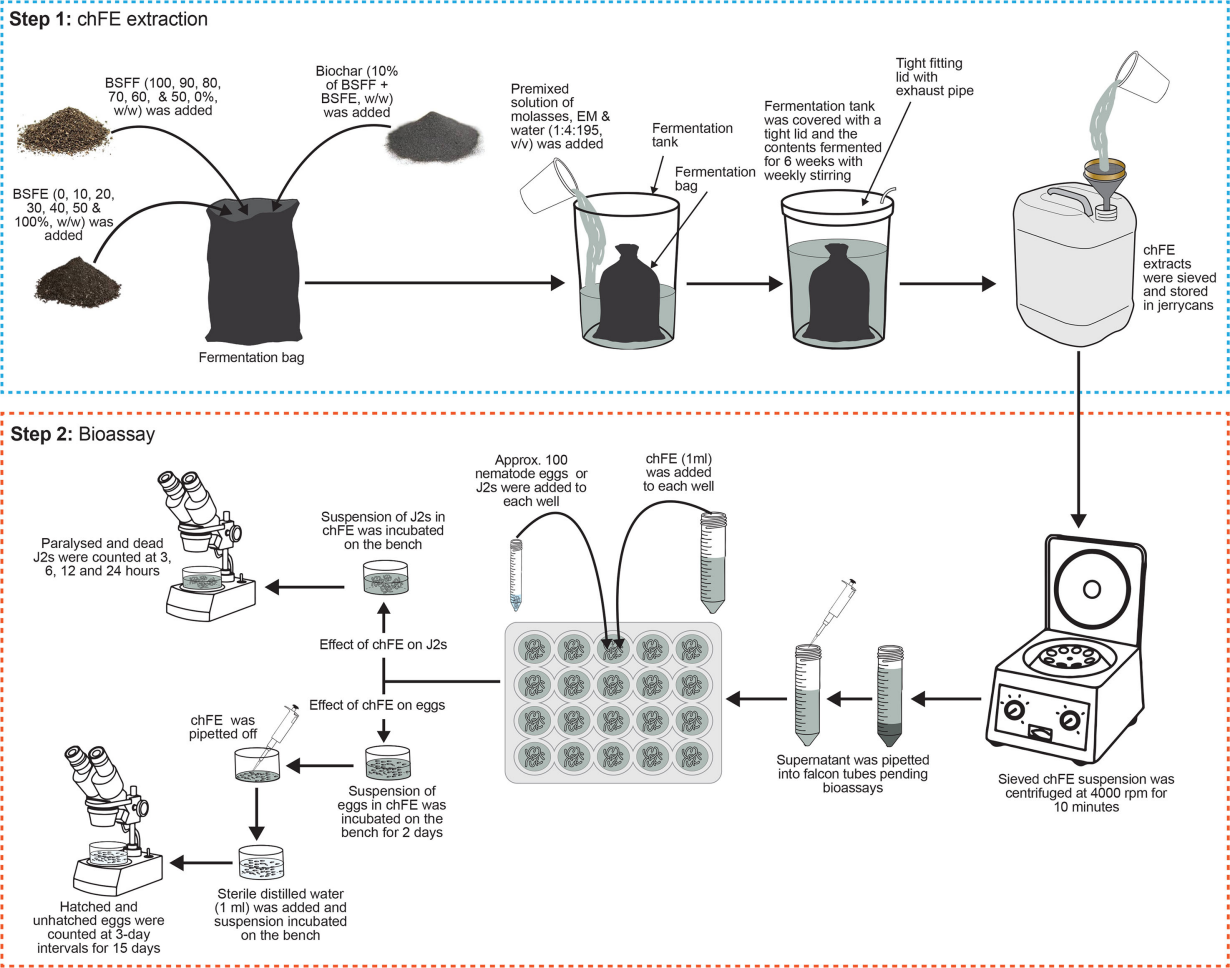
Schematic presentation of methodology used in preparation of chitin-fortified black soldier fly frass fertilizer extracts (chFE) and bioassays.

The BSFF and BSFE were mixed in varying proportions to form seven treatments with 0%, 1%, 2%, 3%, 4%, 5%, and 10% chitin composition and were denoted as E0, E10, E20, E30, E40, E50 and E100, respectively (**Table 1**). The quantity of chitin was estimated based on its composition in BSFE following biological extraction using *B. subtilis*, *P. aeruginosa*, and *L. plantarum* (Lagat et al., 2021). The mixed solid materials (BSFF, BSFE and biochar) were placed in bags made of charmeuse fabric; the bags measured 35 × 70 cm (diameter × height). The bags were tightly sealed and individually placed in 50-L non-transparent plastic fermentation tanks, and each tank was filled with 40 L of a premixed solution of molasses, EMs and water (1:4:195, v/v/v) (**Table 1**). The tanks were tightly covered with a lid fitted with a 5-mm-diameter tube to allow for gaseous exchange. The contents were fermented for 6 weeks with weekly stirring to obtain mature and stable chFE. The maturity and stability of frass fertilizer extracts were determined following previously described procedures (Bernal et al., 2017; Beesigamukama et al., 2021). After fermentation, the chFE were sieved through a 150-µm sieve (Endecotts Ltd., London, UK), put into 20-L non-transparent jerrycans, and stored in a cool place pending bioassay experiments. Prior to use in the bioassay experiments, approximately 45 mL of chFE was placed in 50-mL Falcon tubes and centrifuged at 4,000 rpm for 10 minutes. The supernatant was collected into 50-mL Falcon tubes ready for use in the bioassays (**Figure 1**).

**Table 1:** Composition of treatments used in the composting and extraction of chitin-fortified frass fertilizer extracts (chFE).

chFE treatment	BSFF (kg)	BSFE (kg)	Biochar (kg)	Molasses (L)	EMs (L)	Tap water (L)
E0	7.2	0	0.8	0.2	0.8	39
E10	6.48	0.72	0.8	0.2	0.8	39
E20	5.76	1.44	0.8	0.2	0.8	39
E30	5.04	2.16	0.8	0.2	0.8	39
E40	4.32	2.88	0.8	0.2	0.8	39
E50	3.60	3.60	0.8	0.2	0.8	39
E100	0	7.20	0.8	0.2	0.8	39

BSFF, black soldier fly frass; BSFE, black soldier fly exuviae; EMs, effective microorganisms.

### 2.2 Nematode eggs and infective juveniles

*Meloidogyne* sp. nematodes were isolated from infested tomato roots collected from the National Youth Service (NYS), Yatta Agricultural Farm, Machakos, Kenya. Single egg masses were picked from the galled tomato roots using a stereo microscope (Leica M80, Leica Microsystems, Teban Gardens Crescent, Singapore) and used to establish pure cultures of *M. incognita*, which were used in this study. The identity of *M. incognita* was established using the perennial pattern of mature females (Taylon et al., 1956). Prior to use in this study, the nematode inoculum was multiplied on tomato plants (cv. money maker) in 2-L pots containing steam-sterilized soil in the screen house at *icipe*, Nairobi, Kenya.

To generate nematode eggs, heavily galled tomato plants (approx. 2 months old) were uprooted, and the roots were gently washed with tap water and cut into *ca*. 1-cm pieces. The cut root pieces were then placed in a 300-mL conical flask and completely covered with 0.6% (w/v) sodium hypochlorite (NaOCl) solution. The conical flask was shaken vigorously for 5 minutes to dissolve the gelatinous matrix and free the eggs. The contents of the conical flask were then poured through a 250-µm sieve and placed over a 25-µm sieve (Endecotts Ltd.). The collected eggs on the 25-µm sieve were quickly washed with running tap water to remove all the NaOCl, with a final rinse performed using sterile distilled water, and the eggs were collected in 15-mL Falcon tubes (Coyne and Ross, 2014) ready for use in the egg hatchability assay. A portion of the collected eggs were hatched using the modified Baermann funnel technique (Hallmann and Subbotin, 2018) at 25°C–27°C for 5–7 days; the hatched J2s were used in the *in vitro* and *in planta* bioassays and the assessment of infective juvenile paralysis and mortality.

### 2.3 *In vitro* bioassays

#### 2.3.1 Egg hatching assay

Prior to assessing the antagonism of chFE against *M. incognita* eggs, the nematode eggs were quantified using a stereo microscope, and their density was adjusted to 1,000 eggs per mL. A 10% dilution of each chFE was prepared using sterile distilled water, and 1 mL of each dilution was separately transferred into each well of the 24 multi-cell culture plates (Thermo Fisher Scientific, Waltham, MA, USA). Velum^®^ Prime (Bayer Crop Science, Cambridge, UK) at a volume of 1 mL, diluted as per the manufacturer’s recommendation, was used as a commercial nematicide, while sterile distilled water was used as the control. Approximately 100 *M. incognita* eggs, contained in 0.1 mL suspension, were added to each well. The eggs were incubated in the treatments for 48 hours; thereafter, the treatment solution was carefully pipetted off. Pipetting of the treatment solution was performed under a stereo microscope, and care was taken not to suck up any eggs or hatched nematodes. Thereafter, sterile distilled water (1 mL) was added to each well, and the eggs were incubated on the laboratory bench for 15 days. Twenty replicate wells were set up for each treatment, and the number of emerged J2s was counted at 3-day intervals for 15 days. At each time point, four wells were randomly selected from each treatment, the contents of the wells were transferred onto a 2-mL De Grisse counting dish (De Grisse, 1963), and the number of hatched and unhatched eggs was quantified using a stereo microscope (**Figure 1**). The experiment was repeated once in time.

#### 2.3.2 *M. incognita* infective juvenile paralysis and mortality assay

Approximately 100 freshly hatched J2 suspension (0.1 mL) was transferred into each well of the 24 multi-cell culture plates, and either diluted chFE or commercial nematicide (1 mL) was added. Sterile distilled water was used as the control. Each treatment was replicated 16 times, and the setup was incubated on the laboratory bench for 24 hours in a completely randomized design. Nematode paralysis and mortality were assessed at 3 hours, 6 hours, 12 hours and 24 hours post treatment application. At each time point, four replicates of the same treatment were randomly selected, and the nematodes were washed with sterile distilled water and then assessed for paralysis. Nematodes were recorded as paralyzed if they appeared straight and non-motile. A similar bioassay was set up to assess nematode mortality, where nematodes were considered dead if they failed to move after probing with a fine needle (**Figure 1**). The bioassays were repeated twice.

### 2.4 Nematicidal efficacy in spinach plants

**Figure 2:**
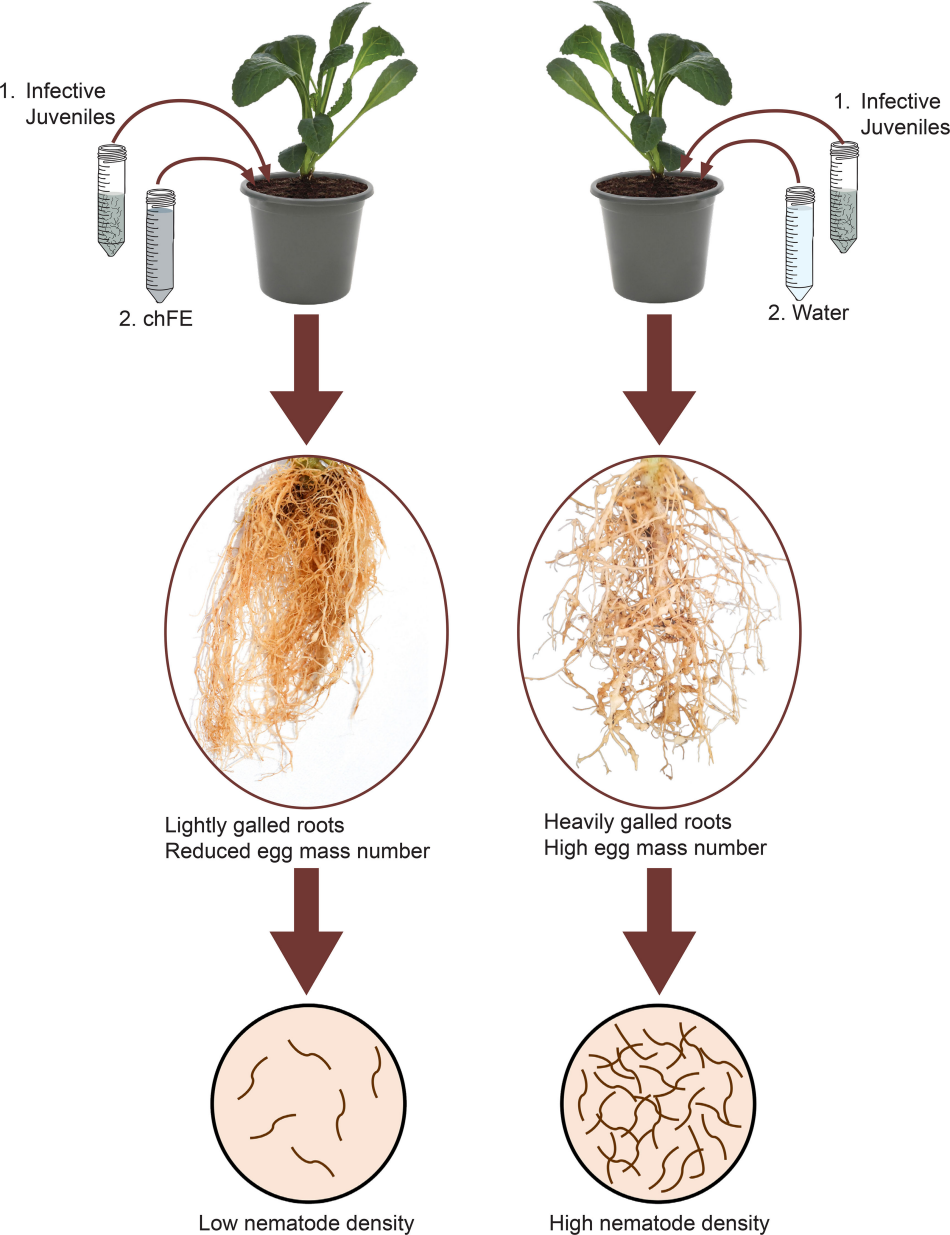
Schematic presentation of methodology used in *in planta* bioassay used to assess the efficacy of chitin-fortified black soldier fly frass fertilizer extracts (chFE) against *Meloidogyne incognita* infection of spinach plants.

*In planta* efficacy of chFE was evaluated using spinach plants (cv. Fordhook giant). Four-week-old seedlings were individually transplanted into 2-L pots filled with a steam-sterilized potting mixture of loam soil and fine sand (3:1). The pots were maintained in a greenhouse. One week after transplanting, each pot was inoculated with a 2 mL suspension of *M. incognita* (1,000 J2s) (Coyne and Ross, 2014). One day after infection with J2s, three dilutions (25%, 50% and 100%; v/v) of each chFE were prepared. Each pot was treated by drenching with 100 mL of either chFE or commercial nematicide. Commercial nematicide was diluted as per the manufacturer’s guidelines, while control plants were drenched with tap water (**Figure 2**). Six replicate pots were used for each treatment, with the treatments arranged in a completely randomized design, and the experiment was conducted twice. The treatments were applied biweekly, and the experiment was terminated 7 weeks after nematode inoculation.

At experiment termination, the plants were carefully removed from the pots, and the roots were gently rinsed using tap water to remove all adhering soil and then blotted dry using tissue paper. The roots were cut from the shoot, and the root and shoot fresh weights were recorded. The roots were scored for root galling using the index described by Coyne and Ross (2014); furthermore, the number of galls per plant root system was recorded. The egg masses were stained by soaking the roots in phloxin B solution (0.1%) for 15 minutes, followed by rinsing in tap water. The egg masses were counted, the roots were chopped into *ca*. 1-cm pieces, and J2s were extracted using the modified Baerman techniques (Coyne et al., 2018b) over a 14-day period. Nematode suspensions were collected from the extraction plates once every 2 days and kept in the fridge (4°C–6°C), and fresh water was added to the extraction plate. For each treatment replicate, the extracted J2s were consolidated over the 14-day extraction period prior to quantification.

### 2.5 Data analysis

Due to the binary nature of egg hatchability data (hatched *vs.* unhatched) and nematode paralysis data (paralyzed *vs.* non-paralyzed), the data were fitted using generalized linear models (GLMs) with binomial distribution to determine the main effects of experiment repeat, treatment, exposure time, and the interaction effects. The Kaplan–Meier survival analysis (log-rank method) was performed on nematode mortality data using the R package “survival” (Therneau, 2022) to assess if the proportion of dead J2s was influenced by experiment repeat, treatment, and exposure time. At 24 hours post exposure of J2s to treatment, the proportion of dead nematodes was fitted using a GLM with binomial distribution to check for the ultimate effect of treatment on nematode mortality. Median lethal times (LT_50_) were determined using the “dose.p” function of the package “MASS” (Brian et al., 2021). LT_50_ values were determined only for treatment repeats that yielded >50% nematode mortality. The LT_50_ values were fitted using a GLM with Gaussian distribution to check for the effect of treatment.

In the experiment to assess *in planta* efficacy of chFE against *M. incognita* infection of spinach plants, data on plant growth (shoot and root fresh weight) were subjected to analysis of variance (ANOVA) to investigate the main and interaction effects of experiment repeat, chFE and concentration treatments. Prior to ANOVA, data on root weight were square root-transformed to conform to the requirements for normality (Shapiro and Wilk, 1965) and homogeneity of variances (O’Neill and Mathews, 2002). No transformation was performed on shoot weight data. Data on galling index, number of galls, egg masses and J2s per plant root system were fitted to GLM with Gaussian distribution to check the effects and interactions between experiment repeat, chFE and concentration treatments. The significance of the GLM models was established using an analysis of deviance (Wald chi-square test) followed by computation of least-square means using the “emmeans” package (Lenth, 2022), and group means were separated using Tukey’s [honestly significant difference (HSD)] multiple comparisons using the “cld” function of the “multcomp” package (Hothorn et al., 2021). All data analyses were performed using R (Version 4.2.2) statistical software (R Core Team, 2022), with statistical significance noted at p < 0.05.

## 3 Results

### 3.1 Effect of chitin-fortified frass fertilizer extracts on egg hatchability

**Figure 3:**
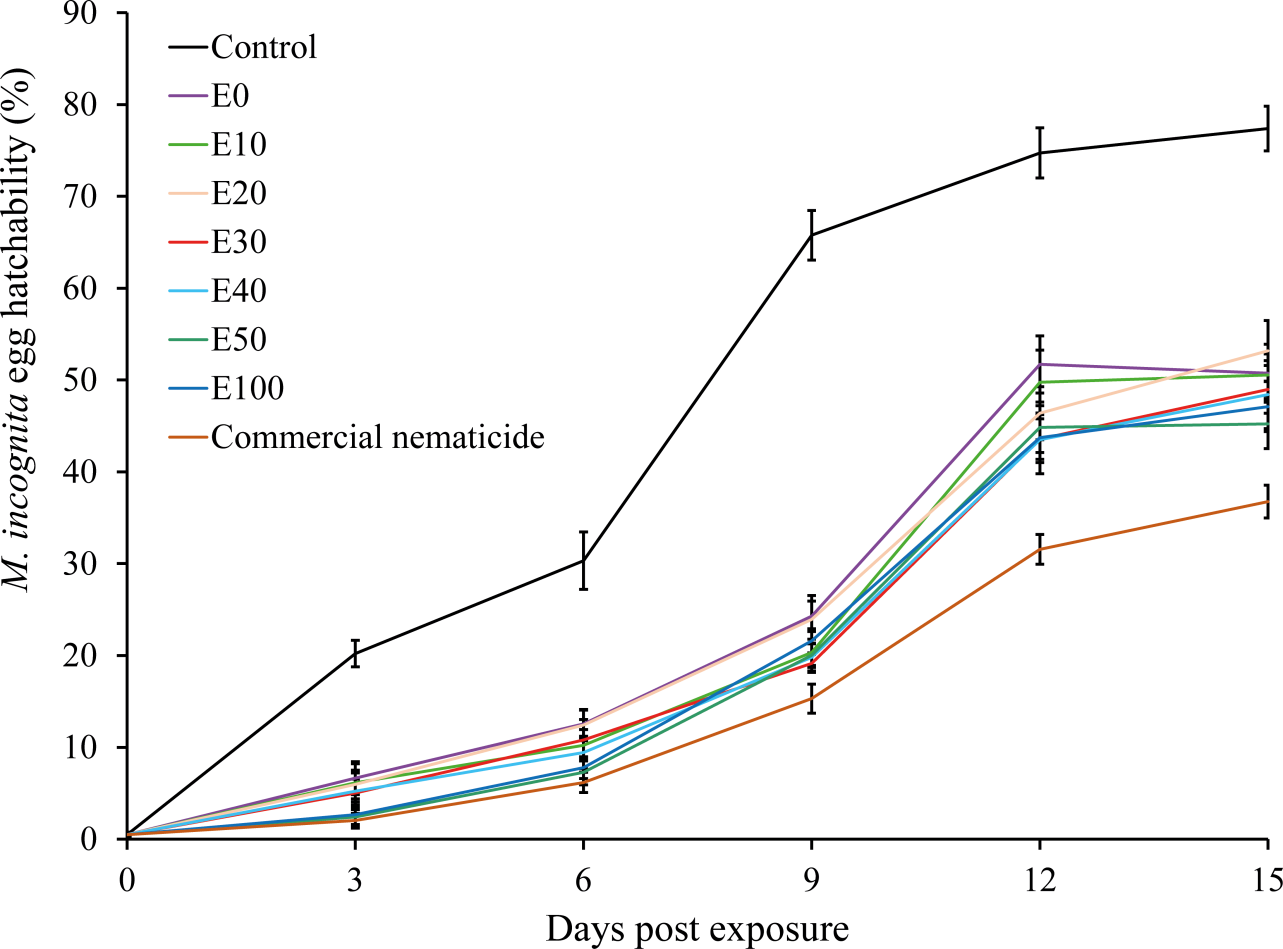
Suppression of hatchability of *Meloidogyne incognita* eggs upon exposure to chitin-fortified frass fertilizer extracts for 48 hours and incubation in sterile distilled water over a 15-day period. Data pooled across two experiments.

Overall, the proportion of hatched eggs in experiment 2 (53%) was significantly higher than in experiment 1 (49%). Similarly, egg hatchability was significantly influenced by treatment and time of exposure. Apart from the significant interaction recorded between treatment and time of exposure, all other interactions were non-significant. Consequently, data were pooled across experiment repeats prior to further analysis. Exposure of nematode eggs to commercial nematicide and chFE significantly inhibited egg hatchability compared to the control. Furthermore, the proportion of hatched eggs increased with time across all treatments (**Figure 3**).

**Figure 4:**
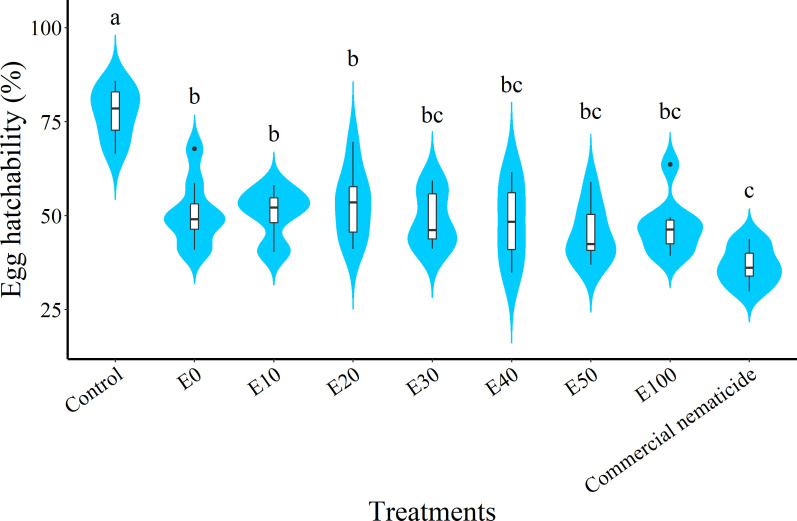
Violin plots showing suppression of *Meloidogyne incognita* egg hatch at 15 days post exposure to chitin-fortified frass fertilizer extracts. Plots followed by the same letter(s) are not significantly different. Means separated by Tukey’s [honestly significant difference (HSD)] test at p < 0.05. Data pooled across two experiments.

At day 15 post treatment, the suppression in egg hatchability achieved by chFE (47%–55%) and commercial nematicide (63%) was significantly higher than that in the control (23%) (**Figure 4**). However, the chFE E30, E40, E50 and E100 exhibited suppression of nematode egg hatchability like that of commercial nematicide (**Figure 3**).

### 3.2 Effect of chitin-fortified frass fertilizer extracts on paralysis of *M. incognita* infective juveniles

All the seven chFE products and the commercial nematicide paralyzed *M. incognita* J2s. Percentage nematode paralysis was not influenced by experiment repeat. However, this varied across treatments and time of exposure, with a significant interaction between treatment and exposure time. Consequently, data were pooled across experiments prior to further analysis. Overall, nematode paralysis increased with increased exposure to the treatment. Similarly, nematode paralysis was significantly higher in the chFE and commercial nematicide treatments compared to the control at each of the four time points. While the commercial nematicide had significantly higher nematode paralysis at 6 and 12 hours compared to chFE, this was not observed at 3 and 24 hours post exposure (**Table 2**).

**Table 2:** Percentage paralysis of *Meloidogyne incognita* infective juveniles at 3 hours, 6 hours, 12 hours and 24 hours post exposure to chitin-fortified frass fertilizer extracts (chFE).

chFE treatment	Time post exposure (hours)			
	3	6	12	24
Control	2.1 ± 0.5 bB	3.1 ± 0.4 cB	3.2 ± 1.0 fB	10.8 ± 0.9 bA
E0	37.8 ± 1.9 aC	41.7 ± 2.1 bC	79.4 ± 5.3 deB	98.8 ± 0.4 aA
E10	36.9 ± 2.3 aD	47.0 ± 2.3 bC	86.2 ± 0.9 cdB	99.6 ± 0.2 aA
E20	37.7 ± 1.9 aC	43.3 ± 1.8 bC	90.3 ± 2.5 bcB	99.6 ± 0.3 aA
E30	32.2 ± 3.0 aD	44.5 ± 1.1 bC	92.8 ± 2.9 abB	99.6 ± 0.3 aA
E40	38.2 ± 1.9 aC	46.5 ± 5.8 bC	75.8 ± 2.6 eB	99.3 ± 0.3 aA
E50	36.1 ± 3.3 aC	42.4 ± 3.1 bC	84.2 ± 0.8 cdeB	99.2 ± 0.3 aA
E100	40.5 ± 1.3 aC	41.0 ± 2.4 bC	91.8 ± 0.9 bcB	99.6 ± 0.3 aA
Commercial nematicide	43.7 ± 2.3 aC	59.4 ± 1.5 aB	98.8 ± 0.4 aA	99.9 ± 0.1 aA

Values represent mean ± standard error. At each time point, means followed by the same lowercase letter(s) indicate no significant difference. For each treatment, means followed by the same uppercase letter indicate no significant difference. Means separated by Tukey’s [honestly significant difference (HSD)] at p < 0.05. Data pooled across two experiments.

### 3.3 Nematicidal effect of chitin-fortified frass fertilizer extracts on *M. incognita* infective juveniles

**Figure 5:**
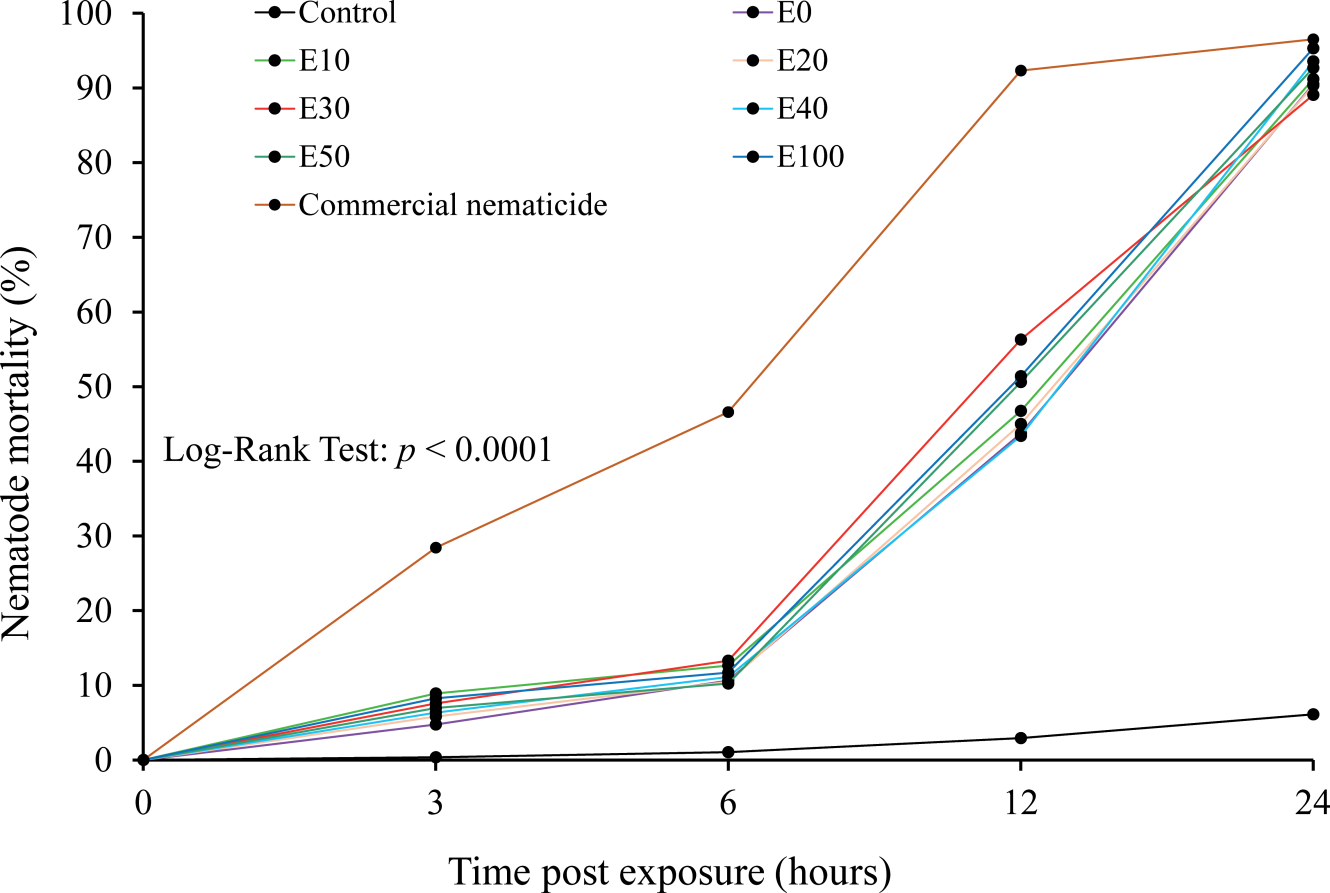
Cumulative mortality of *Meloidogyne incognita* infective juveniles (J2s) at 3 hours, 6 hours, 12 hours and 24 hours post exposure to chitin-fortified frass fertilizer extracts.

The chFE possessed antagonistic activity against *M. incognita* J2s, which varied with time. As time progressed, the proportion of dead nematodes increased with a significant difference across the treatments (**Figure 5**). The mortality rate for the chFE-treated nematodes was higher than that of the control but lower than that of the commercial nematicide.

**Figure 6:**
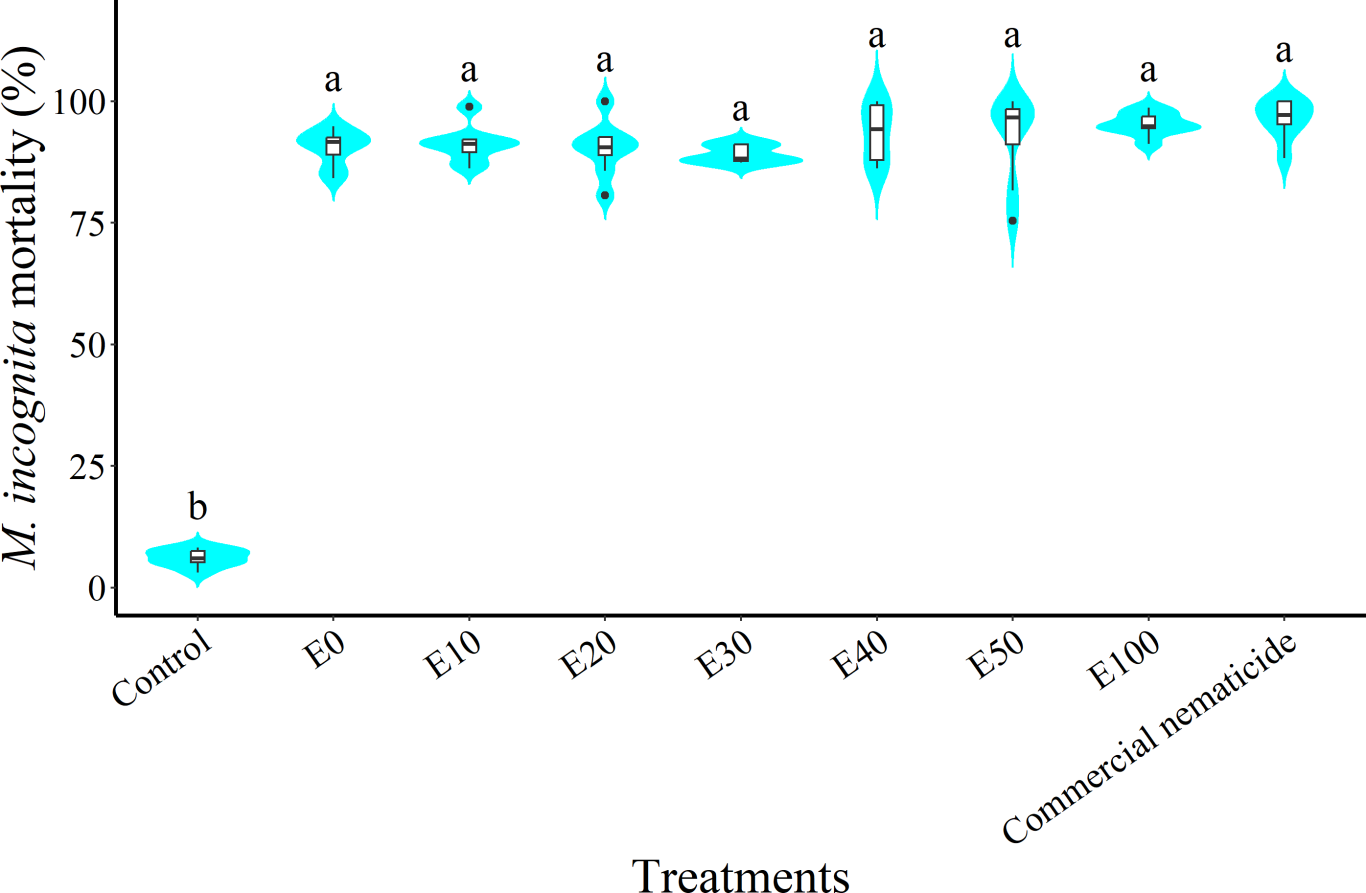
Violin plots showing percentage mortality of *Meloidogyne incognita* infective juveniles (J2s) at 24 hours post exposure to chitin-fortified frass fertilizer extracts. Plots followed by the same letter indicate no significant difference. Means separated by Tukey’s [honestly significant difference (HSD)] test at p < 0.05. Data pooled across two experiments.

At 24 hours post nematode exposure to treatment, nematode mortality did not vary between experiments; however, there was a significant effect of treatment but no interaction of experiment and treatment. Nematode mortality due to chFE was 15–16-fold higher than that of the control and statistically similar to that of the commercial nematicide (**Figure 6**).

**Figure 7:**
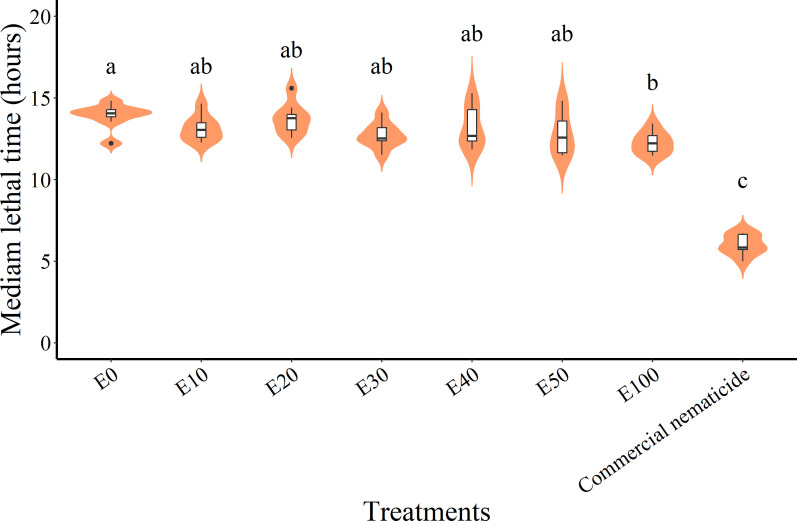
Violin plots describing median lethal time (LT_50_) of *Meloidogyne incognita* infective juveniles (J2s) exposed to chitin-fortified frass fertilizer extracts. Plots with same letter(s) indicate no significant difference. Means separated by Tukey’s [honestly significant difference (HSD)] test at p < 0.05. Data pooled across two experiments.

While the median lethal time (LT_50_) in experiment 1 (11.99 hours) was significantly lower than in experiment 2 (12.47 hours) and was significantly influenced by treatment, there was no significant interaction between the experiment and treatment. Thus, LT_50_ data were pooled across experiments prior to further analysis. Overall, the commercial nematicide recorded the least LT_50_ (6.01 hours), which was lower than that of chFE. In contrast, among the chFE products, E100 recorded the lowest LT_50_ (12.26 hours), which was significantly lower than that of E0 (13.91 hours) (**Figure 7**). Nematode mortality in the control was <50%; hence, no LT_50_ values were estimated.

### 3.4 Efficacy of chitin-fortified frass fertilizer extracts in spinach plants

#### 3.4.1 Plant growth

There was no significant effect of the experiment repeat on either root weight or shoot weight. Apart from E100, drenching the soil with chFE at a 25% dilution rate significantly boosted spinach plant root growth by ≥57% compared to untreated control. Similarly, soil drenching with E0 and E10 at either 25% or 50% dilution rates significantly improved spinach shoot weight by ≥42% relative to untreated control. At dilution rates of 25% and 50%, chFE application significantly increased spinach root (E0, E10 and E20) and shoot (E0, E10 and E30) biomass by 54%–74% and 39%–58%, respectively, compared to the commercial nematicide (**Table 3**).

**Table 3:** Growth of spinach plants cv. Fordhook giant at 7 weeks post infection with 1000 *Meloidogyne incognita* infective juveniles and drenching with chitin-fortified frass fertilizer extracts (chFE) at 25, 50 and 100% dilution rates.

chFE treatment	Dilution rate (%)	Fresh root weight (g)	Fresh shoot weight (g)
Control		1.52 ± 0.07 efg	21.4 ± 0.6 defg
Commercial nematicide		1.53 ± 0.10 efg	21.2 ± 1.0 efg
E0	25	2.66 ± 0.08 a	33.4 ± 1.6 a
E10		2.58 ± 0.08 a	30.4 ± 1.7 abc
E20		2.51 ± 0.09 a	29.4 ± 1.3 abcde
E30		2.45 ± 0.1 abc	29.5 ± 2.8 abcd
E40		2.46 ± 0.11 ab	28.4 ± 1.6 abcdef
E50		2.39 ± 0.12 abcd	26.8 ± 1.1 abcdefg
E100		1.50 ± 0.06 efg	28.1 ± 0.9 abcdefg
E0	50	2.35 ± 0.20 abcd	29.8 ± 2.1 abc
E10		2.40 ± 0.18 abc	31.8 ± 1.6 ab
E20		2.01 ± 0.13 abcdef	27.9 ± 1.3 abcdefg
E30		2.10 ± 0.11 abcde	28.3 ± 2.7 abcdef
E40		1.97 ± 0.13 abcdef	25.2 ± 0.8 bcdefg
E50		1.76 ± 0.12 bcdefg	24.8 ± 1.3 bcdefg
E100		1.74 ± 0.11 cdefg	24.2 ± 2.2 bcdefg
E0	100	1.69 ± 0.17 defg	25.7 ± 0.8 abcdefg
E10		1.57 ± 0.25 efg	24.5 ± 1.4 bcdefg
E20		1.25 ± 0.19 g	24.0 ± 1.8 bcdefg
E30		1.34 ± 0.2 fg	23.6 ± 0.8 cdefg
E40		1.21 ± 0.16 g	20.7 ± 1.5 fg
E50		1.20 ± 0.13 g	20.1 ± 1.9 g
E100		1.18 ± 0.06 g	19.9 ± 1.4 g

Values represent mean ± standard error. Within each column, values followed by different letter(s) indicate significant differences. Means separated by Tukey’s [honestly significant difference (HSD)] test at p < 0.05. Data pooled across two experiments.

#### 3.4.2 Nematode infection

**Figure 8:**
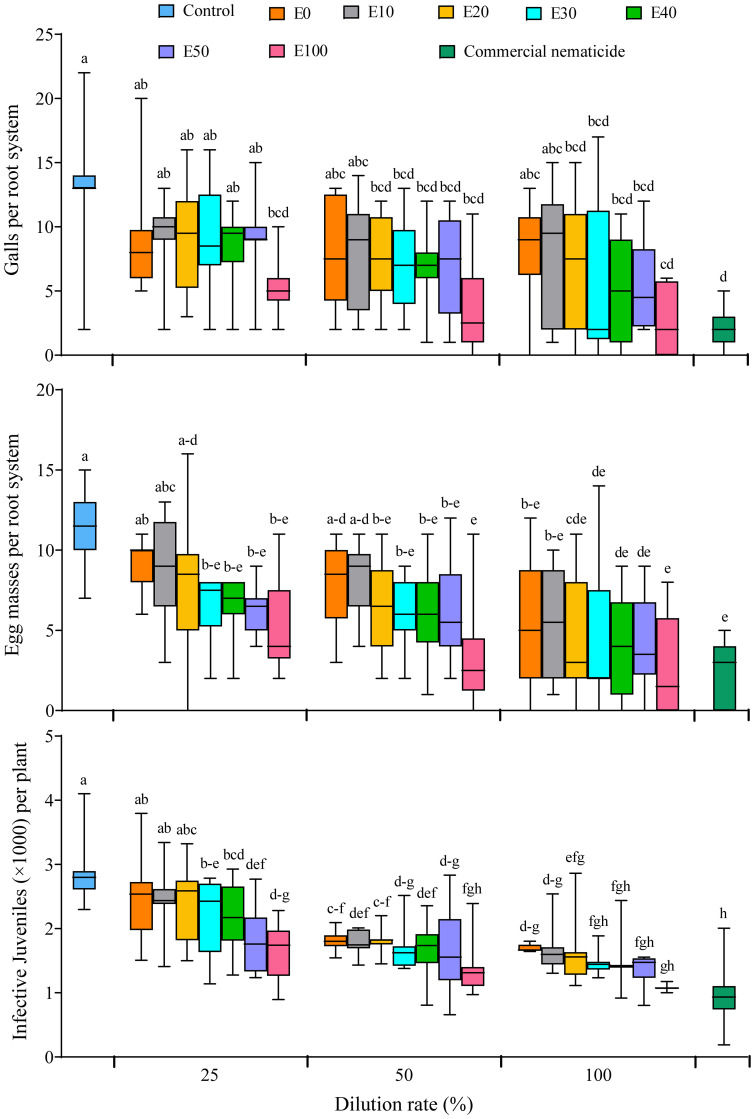
Boxplots illustrating nematicidal effect of chitin-fortified frass fertilizer extracts (chFE) on *Meloidogyne incognita* infection of spinach plants cv. Fordhook giant at 7 weeks post infection with 1,000 infective juveniles. chFE applied at 25%, 50% and 100% dilution rates. For each parameter, boxplots with different letter(s) indicate significant differences at p < 0.05. Data pooled across two experiments (n = 12). Bar in each box indicates the median, while bars at the extremes of the boxes indicate the 25th–75th percentiles. The ends of box whiskers indicate minimum and maximum of the data points.

All the nematode infection parameters (galling index, number of galls, egg masses and infective juveniles) were not significantly influenced by experiment repeat. Similarly, the galling index was not influenced by treatment. However, soil drenching with E20, E30, E40, E50 and E100 at 50% and 100% dilution rates significantly suppressed gall formation in spinach plants by 44%–79%, which was not significantly different from the suppression achieved by the commercial nematicide (85%) (**Figure 8**). Apart from plants grown in soil drenched with E0 and E10 at 25% and 50% dilution, or E20 at 25% dilution, all other chFE treatments significantly suppressed egg mass numbers by 43%–75%, which was similar to suppression achieved by commercial nematicide (79%) (**Figure 8**). Save for nematode densities from plants grown in soil drenched with E0, E10 and E20 at 25% dilution (≥2,416 J2s per plant), the number of nematodes (≤2,196 J2s per plant) recovered from roots of plants grown in soil drenched other chFE treatments and dilutions was significantly lower than that of control plants (2,384 J2s per plant) (**Figure 8**).

## 4 Discussion

In this study, we present the first report demonstrating the potential of using microbially extracted chitin from BSF pupal exuviae in the management of the RKN *M. incognita*. In our study, all the tested chFE inhibited *M. incognita* egg hatchability, achieving up to 42% reduction in hatchability at 15 days post exposure, relative to control. Similar to our findings, inhibition of RKN egg hatchability upon exposure to chitin or its derivatives has been reported (Fan et al., 2019; Zhan et al., 2021) and indicates the potential of insect-derived chitin as a sustainable alternative for nematode management.

The mechanism of egg hatch inhibition by chitin may be related to the influx of toxic compounds through the nematode eggshell upon contact with chitin contained in the chFE or the deterioration of the nematode eggshell leading to *in situ* mortality of the unhatched juveniles (Mota and dos Santos, 2016; Göze Özdemir et al., 2022). In our study, we observed no difference in the inhibition of egg hatchability between the commercial nematicide and the chFE E30, E40, E50 and E100 at 15 days post exposure, an indication that chFE with ≥3% chitin composition is optimal for suppressing *M. incognita* egg hatchability, and thus, chFE could be a potential and environmentally friendly alternative to synthetic nematicides.

While antagonism of the chFE slightly increased with an increase in the proportion of BSFE inclusion, the chFE product E0, which was extracted from BSFF alone, also suppressed egg hatchability and caused both paralysis and mortality of J2s. These findings align with previous studies that have reported the antimicrobial and pesticidal activities of BSFF (Bloukounon-Goubalan et al., 2021; Lagat et al., 2021; Abiya et al., 2022; Barragán-Fonseca et al., 2022; Kemboi et al., 2022). Furthermore, the observed antagonism of E0 could be attributed to the fact that the BSFF is not completely devoid of BSFE; hence, the residual BSFE could have broken down into chitin during fermentation, thus causing the antagonism. Additionally, the biological breakdown of organic matter produces nitrogen, especially ammonium nitrogen (Beesigamukama et al., 2020a, 2021), which has been shown to contribute to the nematode suppressiveness of both solid and liquid composts (Oka and Yermiyahu, 2002).

Thus, in the long run, the use of chFE would be a more sustainable and cost-effective intervention for *M. incognita* management. Nevertheless, the LT_50_ (12.26 hours) exhibited by E100 compared to other chFE (LT_50_ ≥ 12.76 hours) is an indication of the role played by chitin in improving the *in vitro* nematicidal potential of chFE.

Overall, while the commercial nematicide had the shortest median lethal time, killing 50% of the J2s in 6 hours, it should be noted that chFE is a multipurpose product. In fact, apart from E100, our study revealed that chFE, applied at a 25% dilution rate, improved root growth and development of spinach in pots. Furthermore, irrespective of the dilution rate, root and shoot biomass increased with an increasing proportion of BSFF. Previous studies have demonstrated the potential of chFE to improve soil fertility and boost crop yield and soil microbiota (Quilliam et al., 2020; Beesigamukama et al., 2020b; Abiya et al., 2022).

The potential of chitin to suppress nematode infection in plants has been reported for RKNs (Ladner et al., 2008; Eberlein and Westphal, 2023) and other plant-parasitic nematodes (Tian et al., 2000). Infection of plant roots by RKNs is characterized by the development of multinucleate-feeding cells, which enlarge to form galls, with a single gall often feeding one or multiple growing females (Abad et al., 2010; Jones and Goto, 2011). In our study, we observed a significant suppression in gall formation upon drenching the spinach plants with chFE; this is an indication of the failure by J2 to either infect or establish a feeding site in the roots of chFE-drenched plants. In fact, the reported *in vitro* mortality of J2 (≥90%) upon exposure to chFE directly translates into a reduction in the available J2 inoculum level for infection and thus the observed reduction in gall formation, egg mass development and number of J2.

In addition to catalyzing the biological breakdown of BSFE and BSFF into chFE, the microbes used possess additional benefits. For example, *B. subtilis* and P. aeruginosa have been shown to possess antagonism against key plant pathogens and pests including plant-parasitic nematodes (Basyony and Abo-Zaid, 2018; Mazzuchelli et al., 2020) and play a crucial role in improving plant growth and development. Thus, future studies will be necessary to determine the exact mechanisms involved in the nematicidal and plant growth promotion activities of chFE to generate key information necessary for developing chFE-based strategies and products for *M. incognita* control.

Infection of a host plant root by J2 largely relies on the intensity and frequency of the nematode stylet thrusts (movements), which leads to the weakening of the epidermal and subepidermal cell walls, release of secretions, and establishment of a feeding site composed of multinucleate giant cells (Wyss et al., 1992). The observed reduction in *M. incognita* infection of chFE-treated spinach plants can be attributed to the paralysis of J2 upon exposure to chFE under *in vitro* conditions. Paralyzed J2 is characterized by a reduction in the intensity and frequency of stylet thrusts, hence their inability to break through the cell wall and establish a feeding site. Furthermore, nematode paralysis reduces the ability of J2s to move and locate the host plant roots, thus limiting infection to the plant. Although this study focused on the potential of chFE to suppress below-ground nematode infestation, future studies should also assess the efficacy of these products on nematode populations that might have infected the aerial plant parts as reported in the study by Singh et al. (2016).

## 5 Conclusions

The current study has demonstrated the *in vitro* antagonistic effects of chFE against nematode eggs and infective juveniles that were comparable to commercial nematicide. Similarly, chFE exhibited antagonism against *M. incognita* infection of spinach plants through suppression of gall formation, egg mass development and number of J2 in the root system. Thus, chFE could be considered an excellent eco-friendly and sustainable bionematicide for the management of plant-parasitic nematodes to strengthen existing integrated control strategies. Additional research to explore the *in planta* efficacy of chFE in other crops and open-field environments is warranted. Furthermore, future studies to investigate the mechanisms involved in chFE efficacy will help to explore the full potential of chFE for nematode management. The impact of chFE on crop productivity and soil health in terms of crop nutrient uptake and nutritional quality, control of other pests, soil nutrient release and beneficial microbes as well as pathogen suppression should be further investigated.

## Data availability statement

The original contributions presented in the study are included in the article/supplementary material. Further inquiries can be directed to the corresponding authors.

## Author contributions

JK: Conceptualization, Data curation, Formal analysis, Investigation, Methodology, Software, Validation, Visualization, Writing – original draft, Writing – review & editing. DB: Conceptualization, Data curation, Formal analysis, Investigation, Methodology, Software, Validation, Visualization, Writing – original draft, Writing – review & editing. SH: Conceptualization, Methodology, Resources, Supervision, Validation, Visualization, Writing – review & editing. SS: Conceptualization, Project administration, Resources, Writing – review & editing. PT: Conceptualization, Investigation, Methodology, Resources, Writing – review & editing. SK: Funding acquisition, Project administration, Resources, Supervision, Validation, Visualization, Writing – review & editing. CT: Conceptualization, Formal analysis, Funding acquisition, Investigation, Methodology, Project administration, Resources, Supervision, Validation, Visualization, Writing – original draft, Writing – review & editing.
